# Guided meditation for vision acuity training on adolescent myopia: study protocol for an open-label, prospective, multicenter, randomized controlled trial

**DOI:** 10.1186/s13063-021-05922-1

**Published:** 2022-01-06

**Authors:** Yibo Li, Lili Zhu, Raoying Wang, Xingyue Yang, Xinqi Jiang, Tao Lu

**Affiliations:** Beijing University of Chinese Medicine, 11 Beisanhuan Dong Lu, Chaoyang District, Beijing, China

**Keywords:** Simple myopia, Meditation, Vision acuity training, Randomized controlled trial

## Abstract

**Background:**

Currently, the population with myopia climbs steadily, and is developing toward younger age, posing a great concern to the health of adolescents. Myopia in severe cases can cause irreversible consequences such as glaucoma, blindness, and other complications. At present, the solutions for myopia are glasses, medication, and surgery. This study aims to investigate the role of a physiotherapy category based on guided meditation for vision acuity training on adolescent myopia.

**Methods:**

This is a prospective, randomized, multicenter clinical trial. One thousand one hundred forty primary and secondary school students aged 8–18 years old from 27 schools will be recruited and randomly divided into an experimental and a control group at a ratio of 2:1 in two phases, with a training period of 30 days in each phase and a follow-up period of 3 months. No interventions will be conducted during the follow-up period, nor will other interventions employed. Inclusion criteria will meet the diagnostic criteria for simple myopia and −6.00D ≤ spherical lenses ≤ −0.50D and cylindrical lenses ≤1.50D. The primary observation index will be to compare the statistical differences in distant visual acuity between the two groups; the secondary observation indexes will be ocular symptoms (mainly including eye fatigue, dryness, pain, double vision, neck pain, thought disorders, and lags in response), diopter, and astigmatism.

**Discussion:**

The purpose of this two-phase trial is to compare the clinical effectiveness of focused vision-guided meditation with Chinese eye exercises that are also non-pharmacological, non-invasive interventions for myopia, and to maximize the benefit to the subjects. The results will indicate whether the training based on focused vision-guided meditation has the ability to improve distant visual acuity, relieve ocular symptoms, and ameliorate diopter. In addition, this trial will provide clinical efficacy of the training, which is expected to provide meaningful data for vision rehabilitation. At the same time, the vision acuity training method, which is permeated with the concept of Traditional Chinese Medicine (TCM) rehabilitation and health maintenance, will be applied to achieve the goal of preventing or alleviating myopic development and reducing myopia rate.

**Trial registration:**

Chinese Clinical Trial Registry ChiCTR2000038642. Registered on 26 September 2020

## Background

Myopia, also known as short-sightedness or near-sightedness, is a common vision condition in which you can see objects clearly when near to your eye, but obscure when farther away. It occurs when your eyeball is unable to focus precisely, forming images in front instead of right on your retina, resulting in a blurry appearance for distant objects. It is a very common condition that typically starts in childhood. And certain risk factors may increase the likelihood of developing nearsightedness, such as genetics and environmental conditions.

The prevalence of myopia is increasing yearly and has become one of the most serious public health problems worldwide [1]. It is expected that by 2050, 50% of the world’s population will be affected by myopia [2]. The increase in the myopic population is accompanied by an earlier age of onset, usually occurring between 6 and 8 years old, growing rapidly between 13 and 16 years of age, and stabilizing after 16 years old [3]. In countries of East and Southeast Asia, approximately 80 to 90% of urban adolescents have myopia by the time they graduate from high school [1], and China has the second highest number of adolescents with myopia in the world with more than 450 million [4].

Myopia can be divided into simple myopia and pathological myopia. The most common one in adolescents is the simple myopia, which is induced by a combination of genetic and environmental factors, and is related to the weakening of visual regulation caused by spasm of the extraocular and ciliary muscles, which will gradually lead to the growth of the eye axis if it is not restored in time. Therefore, the functions of the extraocular and ciliary muscles are crucial for maintaining good vision [5]. If not corrected in time, myopia will lead to serious and irreversible complications such as retinal detachment, macular degeneration, glaucoma, and blindness [6]. Strengthening vision protection for adolescents has become an urgent issue that needs to be addressed.

Currently, optical correction by glasses is the main intervention method for simple myopia, but it cannot improve the visual acuity of the naked eye. Drugs such as atropine can improve visual acuity, but once the drug is discontinued, the vision problem will rebound or cause even worsen myopia, along with some side effects such as photophobia, blurred vision, and allergic conjunctivitis [7]. Laser surgery may be complicated by other eye diseases, such as near vision impairment and dry eye, and has age restrictions [8, 9].

Vision acuity training refers to the use of optical, psychological, physical, and other methods to produce a certain cognitive load on the visual system of the eyes (including regulation, convergence, eye movements, and the associated movements between them), in a bid to improve the visual function and visual comfort of the visual system and achieve the goal of improving and repairing visual abnormalities in both eyes. It is considered as a purely physical, non-invasive, non-drug therapy with no side effects to relieve eye fatigue and improve vision, with a long history. The earliest and most widely known is the Bates Vision Acuity Training Method, including eye sunbathing, palm massage, rope games, ruler games, pencil games, etc. But due to the complexity of its training, long cycles, etc., it has not been widely promoted. At present, the common vision training mainly relieves the ciliary muscle tension by repeatedly focusing on the distant visual target, looking at the distance and movement of the image to adjust the function of the extraocular muscles, and through the different light color changes to stimulate the visual cortex, as ways to improve vision and restore to a good visual system.

Defined as a form of training to improve concentration and mood, meditation involves a range of practices such as mindfulness meditation, yoga, tai chi, and qigong [10]. Meditation is however not limited to sitting still; one can be in a meditative state while walking, standing, sitting, lying, or even working [11]. A recent article in the *New England Journal of Medicine* highly valued the contemporary value of meditation as “embarking on a new era in psychosomatic medicine” [12]. In recent years, meditation has attracted widespread attention and in-depth research across the world, mainly focusing on psychological diseases [13, 14], pain [15], sleep disorders [16], chronic disease rehabilitation [17, 18], and other fields. However, due to the long intervention period, complex indicators, and difficulty in objective quantification, it is difficult to fully reflect the effects of meditation.

The training based on guided meditation for vision acuity belongs to a kind of regulation training method in the scope of physiotherapy, which mainly uses audio and video guidance to instruct myopia patients to focus on breathing, associate images, enter a meditation-like state, and specialize the current practice. Meanwhile, the patients could exercise the extraocular and ciliary muscles by incorporating simple actions such as hand-eye coordination with the stimulation of meridian points. In doing so, visual function and comfort can be improved. As this training method is simple and has a short period, it is welcomed by youngsters, and the indicators for evaluating vision acuity are simple, so it is easy to quantify the effect objectively.

Therefore, we will conduct a multicenter, randomized controlled study based on guided meditation for vision training. It is hoped that this study, from the perspective of psychosomatic medicine, can stimulate the self-healing ability in adolescent students under the guidance of external physical operations.

## Methods/design

### Ethics

This trial is designed in accordance with the “Ethical Review of Biomedical Research Involving Human Subjects” issued by the Ministry of Health of the People’s Republic of China. The implementation of this project has been reviewed and approved by the Ethical Review Committee of Beijing University of Chinese Medicine (BUCM) for Taolu (4 June 2020, approval number: 2020 BZYLL0306); Dr. Yaoxian Wang, the vice president of BUCM, is the authorized representative under subtitle ethics approval and consent (Ethical Approval Document-Chinese version and Ethical Approval Document-English version). Written informed consent will be obtained from each subject and his/her guardians.

### Study design

**Fig. 1:**
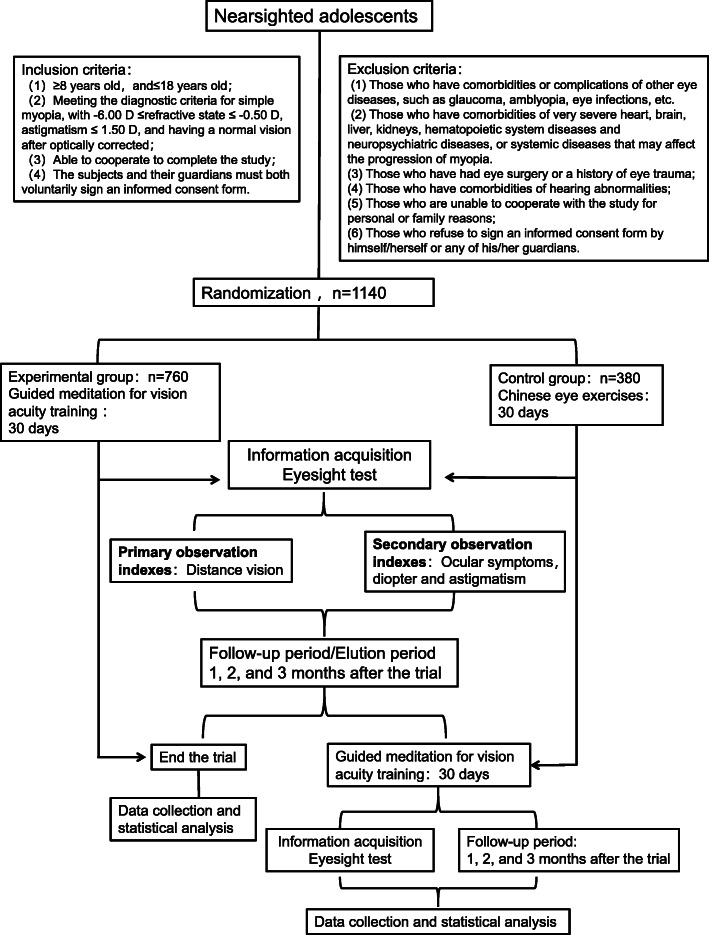
Study design

This study will be a prospective, multicenter, randomized controlled clinical trial. The included subjects (*n* = 1140) will be randomly assigned to the experimental (*n* = 760) and control (*n* = 380) groups in a 2:1 ratio. The aim of this study is to objectively evaluate the clinical efficacy of the guided meditation of vision training for adolescent myopia. This report will be compiled according to the SPIRIT (Standard Protocol Items: Recommendations for Interventional Trials) 2013 Statement “Defining Standard Protocol Items for Clinical Trials” (Fig. 1 and Table 1).

**Table 1:** General procedure and monitoring process

		Study period														
		Enrollment	Allocation	Intervention (phase 1)				Follow-up			Intervention (phase 2)				Termination	Unscheduled visit
Time point		–7~0 d^*^	0 d	1st d^*^	2~7d	8th d^*^	9~30d	M1^*^	M2	M3	1st d	2~7d	8th d	9~30d		
***Enrollment***																
Screening visit		×														
Informed consent		×														
Inclusion and exclusion criteria		×														
History of myopia Intervention		×														
Diet habits		×	×													
Daily activity time distribution		×	×													
CISS^†^		×	×					×	×	×					×	×
Diagnosis		×						×	×	×						
Randomization			×													
Subject files		×						×	×	×						
Adverse events				×	×	×	×	×	×	×	×	×	×	×	×	×
Quit in advance				×	×	×	×				×	×	×	×	×	
Unscheduled visits								×	×	×						×
Allocation			×													
***Interventions***																
Meditation group (phase 1)	The first set of audio and video (1 h)			×		×										
	The second set of audio and video (15–20 min)				×		×									
Control group (phase 1)	Chinese eye exercises			×	×	×	×									
Control group (phase 2)	The first set of audio and video (1 h)										×		×			
	The second set of audio and video (15–20 min)											×		×		
***Assessment***																
Previous visual acuity		×	×													
Current visual acuity		×	×	×	×	×	×	×	×	×	×	×	×	×	×	×
Diopter and astigmatism		×	×				×	×	×	×				×	×	×

*d day, *M* month, *1*^*st*^ *d* the first day, *8*^*th*^ *d* the eight day. †*CISS* Convergence Insufficiency Symptoms Survey

### Recruitment

In this trial, 1140 primary and secondary school students with simple myopia will be recruited from 27 schools in Sichuan and Hunan provinces or other cities with relatively uniform schooling conditions. School factors will be taken into consideration. We will choose participates from different areas with similar teaching level as the subjects. In the meanwhile, a comparison of the experimental group and the control group is launched within the same school in order to reduce the variance.

The main points for the diagnosis of simple myopia in China’s “Myopia Prevention and Treatment Guidelines” [19] will be used as a reference standard for recruitment: near visual acuity is normal; distance visual acuity is less than 1.0 (5.0 by the mucosal method); there are no pathological changes in the fundus; the progression is slow; vision can be corrected to normal with appropriate lenses, while other visual function indicators are mostly normal; and the subject recruited meet the quantitative criteria for myopia according to the “White Paper on Myopia Prevention and Control” of the International Myopia Institute (IMI) [20]: myopia diopter ≤ −0.50D.

### Inclusion criteria

1. ≥8 years old and ≤ 18 years old
2. Meeting the diagnostic criteria for simple myopia, with −6.00 D ≤ refractive state ≤ −0.50 D, astigmatism ≤1.50 D, and having a normal vision after optically corrected
3. Able to cooperate to complete the study
4. The subjects and their guardians must both voluntarily sign an informed consent form

### Exclusion criteria

1. Those who have comorbidities or complications of other eye diseases, such as glaucoma, amblyopia, eye infections, etc.
2. Those who have comorbidities of very severe heart, brain, liver, kidneys, hematopoietic system diseases and neuropsychiatric diseases, or systemic diseases that may affect the progression of myopia
3. Those who have had eye surgery or a history of eye trauma
4. Those who have comorbidities of hearing abnormalities
5. Those who are unable to cooperate with the study for personal or family reasons
6. Those who refuse to sign an informed consent form by himself/herself or any of his/her guardians

### Withdrawal criteria

Subjects have the right to withdraw from the study, as defined in the informed consent document, or to “drop out” if they do not explicitly withdraw from the study but are no longer undergoing training and testing. The reason for withdrawal should be understood as far as possible and recorded, for example, (1) perceiving lack of efficacy, (2) intolerance of certain adverse effects, (3) inability to continue the clinical study, and (4) or lost to follow-up without explanation. Case record forms for withdrawn subjects will be kept, and the results of the last trial will be considered final. The data on the clinical efficacy and adverse effects of the focused vision-guided meditation training will be fully analyzed.

### Suspension criteria

The investigator will decide to terminate the study of a subject who has been enrolled in the study if the subject is unfit to proceed the study. For example, (1) the subject develops certain comorbidities and complications under study; (2) the subject is unfit to continue the study due to unexpected events or specific physio-pathological changes during the study. (3) The subject meets the criteria for cure in less than 30 days; the study may be discontinued early if the subject so desires.

### Excluded cases

Those who with any of the following should be excluded:

1. Subjects with incomplete case information that affects the analysis of the trial results. For subjects, who missed 4 or more days in the first 7 days of treatment, or participated in daily training for less than 14 days. Patients with lost contact were followed up without corresponding prediction and correlation analysis. For research, more than half of the cases in which data was lost due to machine failure or forgetting to record.
2. Subjects with poor compliance during the trial due to subjective or objective factors of their own or family members. Who shows obvious resistance, fall asleep, or play during training and continue to do so after being reminded by the researchers.
3. Subjects who use other interventions for myopia outside the protocol: drug therapy, acupuncture, orthokeratology, etc.

## Randomization

The random sequence will be designed and processed by the research team of Beijing University of Chinese Medicine. SPSS 20.0 (SPSS Inc., Chicago, IL, USA) software will be used to generate serial numbers for the subjects. Multiple factors that affect this trial will be taken into consideration. Participants from different areas with similar school level will be stratified. A stratified randomizing method will also be adopted according to region, age, gender, etc. Similar stratified groups will be compared; the experimental and the control groups are launched within the same school.

As this study will cover 27 schools, a multicenter trial, stratified randomization will be adopted. Firstly, 27 schools will be allotted east, west, north, south, and middle regions of China. Secondly, schools in each district will be divided into 3 layers according to age. Children aged 8–11 years, 12–14 years, and 15–18 years, severally. This method will minimize errors and improve accuracy.

### Allocation concealment

The randomization lists will be kept in opaque envelopes by the non-investigator on the team which are ordered, identical, and sealed with adhesive.

### Implementation

The participants will be consecutively enrolled and distributed into experimental and control groups by researchers according to the randomized sequences generated who will not participate in assessing outcomes. Another group of researchers responsible for analyzing results will be blind to the grouping of subjects and will not participate in the treatment. The executors of this trial and subjects will not be blinded to the treatment assignment.

## Interventions

### Explanation for the choice of comparators

The choice of the comparator is based on the current treatment of myopia in clinical.

### Intervention description

The vision acuity training of this study is conducted in the schools, and school leaders are fully aware of this project, support, and cooperate with the research work. Relevant teachers will be designated by the schools to be responsible for this project with respect to the specific matters concerned. The study will be conducted in two phases over a period of 8 months, with a training period of 30 days in each phase and a follow-up of 3 months. No interventions will be conducted during the treatment period and follow-up period. One visual acuity examination will be performed in each of the two phases at months 1, 2, and 3 of the follow-up.

### Phase 1

#### Experimental group

Two sets of vision acuity training movement verbal commands will be applied in the experimental group. In order to facilitate the subjects to follow the verbal commands in a uniform, standardized way, the related audio and standard movements will be recorded.

The first set of audio and video content are relatively rich, lasting about 1 h, and the audio and video will be projected in the classroom on the first and eighth days of training, with the subjects following the verbal commands and the movement demonstrations in the video. Visual training tutors (trained in procedures and methods) will be responsible for audio and video presentations.

The second set of audio and video content are relatively concise, approximately 15–20 min in length, and the subjects will be trained under the schools’ uniform deployment for the remaining 28 days.

Due to the uninterrupted daily vision acuity training for 30 days, it is recommended to use the last self-study class at the schools to ensure that the students’ normal studies are not interrupted; students will still be required to come to get trained at their school or a designated location confirmed with the schools on Saturday.

#### First training

The first set of audio and video training exercises will be projected in the classroom by the vision acuity training tutor, and the students will be guided by the audio-visual movements and verbal commands to perform the following exercises.

1. Abdominal breathing: Inhale through the nose, exhale through the mouth, bulge abdomen while inhaling, tighten abdomen while exhaling; start doing abdominal breathing when hearing the words of “adjust your breath and inhale.”
2. Eye-warning exercise: Rub your hands more than 30 times, cover your eyes with the palms, place thumbs on the temples, clasp four fingers on your forehead, and leverage the temperature of your palms to warm eyes; after the temperature gradually dissipates, use Yuji acupoints of the palms to gently press your eyes 5 times, with moderate intensity; start eye-warning exercise when you hear the words “get ready to press” and “quickly rub your hands” during training.
3. Healthy vision exercise: Press both hand palms on your ears, place fingers on the back of your head; do not move the palms, and use fingers to gently tap the back of your head. During the training, when you hear “healthy vision exercise,” start doing it.
4. Activate Shixuan acupuncture point: Tap your fingers on your palm, shake your palm, and make an arrow shape.
5. Play the “1-Audio,” turn off the lights in the classroom, close the curtains, and try to create a dark environment. Remain quiet the entire time. Close your eyes until the audio says to open them. Follow the audio to imagine and do movements, e.g., “Use the fingertips of one hand to gently touch the center of the palm of the other, and then shake your hand vigorously.”
6. Play the “C-video,” a video about lotus flowers to stimulate the imagination and match the 2-Audio with the images that appear in the video. For example, “On a calm lake, you can definitely see the vivid color of the lotus flowers and the greenish-blue leaves of the lotus .......”
7. Demonstration and correction. Make a hollow bowl shape with your hands (thumbs on the second knuckle of your index finger) and clasp them over your eyes, with palms facing your eyes.
8. Play “2-Audio” and do the eye stretches: Close your eyes and move them from front to back as if a train is moving back and forth in a tunnel.
9. Play “3-Audio” and listen to the audio with your hands in a hollow bowl shape over your eyes the whole time. When the audio says “in,” put pressure on your eyes for 1 s, and when it says “out,” relax your eyes and perform the related imagery and actions. When the audio says “pull” in future exercises, let your eyes enter a state just like going into a tunnel, hold for 1 s, and then relax.
10. Play “D-Video (Ball of Light)” to stimulate imagination and introduce natural light.
11. Play “4-Audio” to build up the confidence of myopia sufferers to restore their eyesight.
12. The above training will be repeated on the 8th day to intensify the effect, for 1 h.

#### Daily vision acuity training

Guided by the second set of audio and video movement verbal commands, the subjects perform self-rehabilitation exercises.

1. Ocular exercises: keep body and head still, torso upright, move only eyes, four 8-tempos per part.

In the first part, both eyes look up and down to the left. In the second part, both eyes look up and down to the right. In the third part, both eyes look to the left to the right. In the fourth part, both eyes turn clockwise. In the fifth part, turn both eyes counterclockwise. In the sixth part, close your eyes hard and then open them to look up. In the seventh part, close your eyes for a moment to control the time.

(2) Eye-warming exercises: same movements as before, 12 movements per day
(3) Vision acuity exercise: same movements as before, 24 movements per day
(4) Character card stretching: 10 min of training, with results recorded on an observation chart. Character cards (Chinese characters of different sizes) are placed on a well-lit wall at the subject’;s eye level. Find the farthest place where you can see the card clearly, that is, about 10–15 cm back where you cannot see the card clearly. Let your eyes stretch 6 times for 1.5 s each (eye movement, stretching backwards), without blinking, stare at the characters for 10 s, step back 15 cm, and repeat the above movements until you are unable to see the location of the character card after stretching. If the training time is less than 10 min, switch to a second character and repeat the above steps; if it has reached 10 min, end the exercise and take notes.

#### Control group

The control group will be given Chinese eye exercises in the first stage of the trial. On the first day and the eighth day of the trial, the school teachers who have received training in Chinese eye exercises will give instructions on finding the positions of acupoints, massage intensity, direction, and frequency according to the standard Chinese eye exercises Assessment Form to ensure that the students can perform the eye exercises independently and with high quality. For the remaining 28 days, subjects will be in a uniform classroom for 15–20 min of daily Chinese eye exercises, acupoints: Jingming, Zanzhu, Yuyao, Tongziliao, Sizhukong, Taiyang, Chengqi, and Sibai.

#### Second phase

After the completion of the first phase of the randomized controlled study, data will be collected and compared between the vision acuity training group and the Chinese eye exercise group.

The second phase will be carried out based on the experimental results of the first phase. If the first phase showed that the visual training effect was not significantly better than eye exercises, the trial was terminated. If the data proved that the effect of the meditation group was not superior to the control group, and the results were statistically significant, the second phase of the experiment will be carried out.

Since the purpose of this study is to improve the naked eye visual acuity that affects life and work, the experimental group has achieved this effect in the first stage. In order to ensure the maximum benefit of the subjects, only the control group and the experimental group of the first stage were given the same treatment plan in the second stage, namely guided meditation for vision acuity training. The results of phase 2 subjects (the control group at phase 1) were compared with the results of eye exercises at phase 1.

The subjects in this study are adolescent students with simple myopia and no other comorbidities of chronic diseases. If the subjects need to combine medication due to cold or other reasons, they should be recorded promptly.

### Basic information collection

Information will be collected from the subjects before the start and after the trial is completed, and the main information is as follows.

- Basic information: age, nation, grade, height, weight, family history of myopia, parents’ high myopia, etc.
- Myopia: duration of myopia, possible causes of myopia, naked eye visual acuity, corrected visual acuity, diopter of both eyes, astigmatism, and so on
- Time allocation for daily activities: including close-range activities (< 50 cm): such as drawing, homework, reading, and using electronic screen terminals such as mobile phones; outdoor activities: including playing outdoors, cycling, hiking, etc.

### Primary observation indexes

Visual acuity training aims to improve naked eye visual acuity, which affects life and work and will be used as the main observation index. Visual acuity tests were performed before and after each training session for the first 7 days, once a week after 7 days of training and at the first, second, and third months of follow-up, respectively.

The specification for visual acuity testing is based on the “Expert Consensus on Workflow of Myopia Screening in Children and Adolescents (2019)” [21]. The international standard logarithmic visual acuity chart is adopted. During the measurement, the subjects stand 5 m away from the visual acuity chart and cover the contralateral eyes without squinting and oppression. The covered eyes are examined first in the right eye and then in the left eye. If the inspector can identify half or more of the visual markers, check them line by line from top to bottom and ask the subjects to say or gesture the direction of the gap of the visual markers within 5 s. The last line of the visual markers that is correct will be the testee’s vision of that eye. For example, if row 4.8 cannot be identified, check upward until half or more of the rows can be identified.

### Secondary observation indexes

Ocular symptoms, diopter, and astigmatism are secondary indexes. Ocular symptoms will be evaluated before and after 30 days of training, respectively. Diopter will be obtained from the subjects after testing at the same hospital before and after the training.

#### Ocular symptoms

- Ocular symptom scores are developed based on the “Visual Fatigue Test and Evaluation Method (VFTEM)” and principally cover eye fatigue, dryness, pain, visual double vision, neck pain, thought disorders, and lags in response.
- Collective Insufficient Symptoms Survey (CISS): the CISS is a valid tool in quantifying near visual acuity symptoms in adolescents [22] and consists of 15 items, each with five options of varying degrees, scored as follows: never (0), rarely (1), sometimes (2), often (3), and always (4). The sum of the 15 items’ scores (ranging from 0 to 60) is the total score.

All of the above information and scales need to be completed with the help of the subject student’s parents and professional staff.

#### Diopter and astigmatism

The diopter (after pupil dilation by tropicamide) and astigmatism will be obtained from the students after examination at a local first-tier public hospital or public eye hospital accompanied by their family members, and the data will be recorded in the entry of ‘Information Collection - Visual Acuity.”

Categorical variables, such as gender, age, etc., will be compared between groups. In particular, the effects of age on visual acuity will be stratified to reduce the variation.

## Data entry and statistical analysis

### Data collection

A paper version of the case report form will be developed for data collection. The data will be collected using standardized entry terms and structured registration contents as much as possible, which will facilitate post-processing and analysis of the data and minimize the burden on the researcher. According to the original observation records of the subjects, the data manager will load the data into the case report form in a timely, complete, correct, and clear manner. When amending a case, the original record should be clearly visible, and corrections should be signed and dated by the investigator.

### Data entry and storage

The paper-based data should be entered by a dedicated data manager using a dual-track entry to reduce the error rate, and any problems or unexpected matters found in the entry process should be registered and reported promptly so that problems can be dealt with quickly. Observation forms should be subject to random inspections at the end of data entry to understand the quality of the entry and to analyze and address any problems. Once data entry is completed, all original case report information should be kept. The original case report forms, after completion of data entry and verification as required, should be filed in a numbered order, and a dedicated person should be assigned to manage the files and complete the search directory, etc. Also, the entered data should be backed up regularly to prevent loss of data and other unexpected occurrences. Particular attention should be paid to the security of data storage and the protection of the privacy of the subjects and related personnel. All research documents will be stored in special filing cabinets that are locked to ensure the security of the stored documents. In any case, only authorized staff will have access to the data.

## Statistical analysis

### Sample size

The sample size will be estimated based on our early observations [23]. The treatment group was 4.98 ± 0.14, and the control group was 4.92 ± 0.24. Under 20% dropout rate, with the two-tailed *α* = 0.05 and *β* = 0.1 (test efficacy of 0.9), experimental group: control group = 2:1, PASS 15.0 software calculation shows 380 in the experimental group and 190 in the control group. Due to the possible center effect, the final sample size is suggested to be twice expanded. Thus, it is finally confirmed there will be 1140 cases in total, with 760 cases in the experimental group and 380 cases in the control group.

Statisticians suggest that we appropriately expand the sample size to reduce the impact of the central effect on the final results.

### Statistical analysis

Multiple factors that affect this trial will be taken into consideration. Participates from different areas with similar school level will be stratified. A stratified randomizing method will also be adopted according to region, age, gender, etc. Similar stratified groups will be compared; the experimental and the control groups are launched within the same school.

We will use a linear mixed model to analyze incorporating repeated measures. Firstly, visual acuity is independent among subjects and is the primary indicator of observation. Otherwise, it will be measured at the end of each training session and followed up for 3 months. That is important to model the correlation of these repeated measurements.

Statistical analysis using SPSS 20.0 will be performed, and data management software will be used to construct the database. Dedicated staff will be assigned to oversee the management of electronic data, and a clinical research team will be deployed for data entry, validation, reporting, and answering questions. Normally distributed variables will be expressed as their mean and standard deviation (SD), and frequency (percentage) for categorical variables. Baseline characteristics between the intervention and control groups will be compared by performing a two-sample *t*-test and Wilcoxon rank-sum test for continuous variables with and without normal distribution, respectively. All *P*-values are 2-sided and considered statistically significant when less than 0.05.

Primary and secondary observation indexes will be analyzed by the superiority test depending on the treatment, using Per Protocol Set (PPS) and Full Analysis Set (FAS) datasets. The indices of safety evaluation will be analyzed using the Save Set (SS) dataset.

According to the ophthalmic disease efficacy evaluation standard in the Guidelines for Clinical Research of Traditional Chinese Medicine New Drugs:

1. Cure: visual acuity to normal (5.0)
2. Significant effect: visual acuity improved by 4 lines or more with a visual chart
3. Improvement: visual acuity improved by 2 lines or more
4. Ineffective: visual acuity improved by less than 2 rows or decrease

### Missing data

For primary outcome measurements monitored repeatedly, we used the final follow-up time point to calculate the missing rate. All evaluations, in particular the evaluation of the primary outcomes, will be made on the basis of all randomized patients, carry out a primary analysis of all observed data that are valid under a plausible assumption, regardless of whether or not they adhered to the treatment protocol or provided complete data sets.

### Monitoring

A Data Monitoring Committee(DMC)will be instituted during the study, which is consisted of project researchers, ethics committee members, statistical analysis personnel, and implementation teachers, who have no competing interests. It is the responsibility of the DMC to review the trial design and trial documentations prior to the commencement of the study to identify issues that may affect data analysis or patient safety and identify problems during project implementation and take timely intervention measures. The collected data will be checked again at the end of the study.

## Adverse events

In case of any adverse event, subjective discomfort (dizziness, nausea, etc.) or abnormal laboratory parameters of subjects should be treated seriously, the cause should be carefully analyzed, and immediate measures should be taken to maintain the safety of the subject’s life. Procedures: Detailed records should be made on the case report form, and retesting should be performed within 24 h, 7 days, and 14 days as appropriate. Records of persistence, prognosis, and disappearance should be kept.

Treatment of serious adverse events: If there is any serious adverse event in the course of the study, it must be reported immediately to the ethics committee of the organization and the sponsor, and the “Serious Adverse Event Report Form” must be filled; if there is a serious adverse reaction, it should be reported to the National Medical Products Administration within 24 h, and the contact person should be notified according to the telephone number and home address listed in the case report form.

1. Treatment measures: When the subject has an accident or emergency, the investigator should make corresponding treatment according to the experimental therapy and its symptoms and pass the treatment results to the clinical examiner. The institute should record the treatment and results on the case report form and sign it.
2. Follow-up of unmitigated adverse events: all adverse events should be tracked for their cause until they can be appropriately resolved.

## Frequency and plans for auditing trial conduct

The DMC and the ethics committee will meet annually during the trial to review the conduct of the study, Good Clinical Practice (GCP), compliance with the protocol standard operating procedures and applicable regulatory requirements.

## Quality control

Quality control is crucial to the reliability and accuracy of the research results, mainly conducted over the implementation process and data management aspects to achieve quality assurance. Quality control principally covers the following aspects: (1) investigator training: before the implementation of the study, a detailed research protocol and the SOPs should be developed, and participants of the study should be trained and documented; (2) data quality control: investigators should be trained, and the way in which data are collected, the list of all data elements and definitions, and the treatment methods for missing values, invalid entries, incorrect entries, and logically inconsistent data should be clarified during training. To prevent data bias, data entry and statisticians should be masked during the trial. (3) The study will have different teachers assigned to the control and experimental groups in separated training classrooms. So, it ensures that two groups cannot see each other while working on, minimizing the group contamination.

## Confidentiality

This study will fully comply with the relevant provisions of the data protection legislation. All appropriate and necessary precautions will be taken to keep medical data and personal information permanently confidential.

## Discussion

The eyes do not exist in isolation, but are an integral part of what makes up the human body, influencing the body, mind, and emotions in a unique way. Western medical research has shown that the visual system is considerably sensitive to any form of stress, tension, and mental state and that vision problems usually occur along with chronic stress and incorrect eye habits. Nearsightedness, farsightedness, and astigmatism are symptoms that are resulted from this underlying stress and imbalance. A better holistic model of vision includes the following three components: physical eyesight, inner vision, and emotional seeing [24].

The book *Zhu Bing Yuan Hou Lun*, also known as “General Treatise on Etiologies and Manifestations of All Diseases,” from Traditional Chinese Medicine (TCM) states: “The injury to the visceral organs caused by fatigue, deficiency of liver vitality (qi), and exposure to exopathic evil (wind-evil) make the essence of qi or vitality weak, causing vision problems,” pointing out the causalities and characteristics of myopia in a systematic way. The liver is the main reservoir of blood and one of the major sources for vitality, associating strongly with blood capillary supplements for extraocular muscle. Emotion fluctuation as well as excessive use of the eye will compromise liver qi (vitality) or cause it to be stagnant, thereby affecting blood or qi supply to the eye, leading to vision problems. “The Chapter of Discussion on the Generation of Five Zang (visceral) Organs in Su Wen” states: “the heart governs blood vessels, all vessels connect to the eyes.”; the spleen is the origin of the acquired constitution and the source of qi and blood production; if the spleen qi suffers from deficiencies lasted, blood is difficult to nourish eyes, compromising the visual acuity. According to Zhang Jingyue, an ancient TCM master, “lung governs qi, and when qi is coordinated, deficiencies in nutritive qi and protective qi as well as lower vitality in Zang (visceral )-organs are all cured.” The whole body’s blood converges at the lung, and the lung regulates the whole body’s vigor of qi; when the qi of the lung is harmonized, blood can form a steady flow into the eyes, thus benefiting the vision. Thus, the deficiency of essence of visceral organs will dampen the vigor qi for blood flow and make it unable to nourish the eyes, thereby weakening the vision; from the clinical perspective of Chinese medicine, the causalities of vision problems are related to the liver, heart, spleen, lung, and kidney, the five Zang (visceral)–organs [25]. Therefore, vision rehabilitation training should not be limited to the adjustment of the visual system only, but extend to internal and external treatments, as well as physical and physiological regulation holistically.

Meditation is vital for “mental condition adjustment” and “health improvement” in TCM and has been advocated and practiced by medical practitioners in ancient China. It is also one of the populated approaches for self-health management. Meditation is often coordinated with the adjustment of posture, breathing, and consciousness to motivate self-healing capacity. It means to build a peaceful and perceptive mind to overcome the judgmental thoughts or emotions. This helps to eliminate unhealthy muscle memories and psychological preconceptions, potentially creating new ways to cope with illness [26]. Studies have shown that during meditation, parasympathetic activity increases and sensory and perceptual changes occur. These physiological changes are a state of self-healing relaxation that may have preventive and therapeutic effects on human health [27]. Meditation is also a positive mental exercise that alters brain and mind function and facilitates the regulation of attention, cognition, and mood. It is well documented that mental state affects physical condition, and its imbalance affects hormone release, attributing to diseases.

The incorporation of meditation elements into vision acuity training is a multifaceted combination of movement, breathing, and imagination, which may enhance vision by strengthening the eye muscle regulation functions.

This project will be conducted in two phases, using two research designs. The first phase will be a multicenter randomized controlled study, and the second phase will be a single-arm pre-post controlled study. The aim is to compare the differences in the effects of the training based on guided meditation and Chinese eye exercises on vision regulation in adolescents. If the significant clinical efficacy and safety are uncovered, a novel non-pharmacological physical therapy will be provided for the treatment of adolescent myopia. The study will also help to promote and disseminate the vision acuity training method, which is infused with the TCM rehabilitation concept, and provide a reference plan and basis for solving the myopia problem among adolescents.

Certainly, guided meditation for vision acuity training is not always almighty; it also has limitations. For instance, the target population of this method is simple myopia with −6.00 D ≤ refractive state ≤ −0.50 D and astigmatism ≤1.50 D. The research area and sample size also limit its external promotion. Therefore, we will combine meditation with Baduanjin or Taijiquan to form a training of dynamic and static combination in the further research, which could be a better means for myopia of adolescents.

## Trial status

At the time of submission, the formative research has been completed and recruitment is ongoing. The first participant will be enrolled in December 2021. Patient recruitment is estimated to be completed around September 2023. The current protocol is version 3.0 of 30-10-2021.

## Data Availability

The datasets generated or analyzed during the current study are available from the corresponding author upon reasonable request.
